# Clinical Validation of a PCR Assay for the Detection of *EGFR* Mutations in Non–Small-Cell Lung Cancer: Retrospective Testing of Specimens from the EURTAC Trial

**DOI:** 10.1371/journal.pone.0089518

**Published:** 2014-02-25

**Authors:** Susana Benlloch, Maria Luisa Botero, Jordi Beltran-Alamillo, Clara Mayo, Ana Gimenez-Capitán, Itziar de Aguirre, Cristina Queralt, Jose Luis Ramirez, Santiago Ramón y. Cajal, Barbara Klughammer, Mariette Schlegel, Walter Bordogna, David Chen, Guili Zhang, Barbara Kovach, Felice Shieh, John F. Palma, Lin Wu, H. Jeffrey Lawrence, Miquel Taron

**Affiliations:** 1 Pangaea Biotech SL, Barcelona, Spain; 2 Medical Oncology Service-ICO, Hospital Germans Trias i Pujol, Badalona, Spain; 3 F. Hoffmann-La Roche, Basel, Switzerland; 4 Genentech, South San Francisco, California, United States of America; 5 Roche Molecular Systems, Pleasanton, California, United States of America; 6 Pathology Department, Vall d'Hebron, University Hospital, Universidad Autónoma de Barcelona, Barcelona, Spain; 7 Roche Molecular Systems, Pleasanton, California, United States of America; Univesity of Texas Southwestern Medical Center at Dallas, United States of America

## Abstract

The EURTAC trial demonstrated that the tyrosine kinase inhibitor (TKI) erlotinib was superior to chemotherapy as first-line therapy for advanced non-small cell lung cancers (NSCLC) that harbor *EGFR* activating mutations in a predominantly Caucasian population. Based on EURTAC and several Asian trials, anti-*EGFR* TKIs are standard of care for *EGFR* mutation-positive NSCLC. We sought to validate a rapid multiplex *EGFR* mutation assay as a companion diagnostic assay to select patients for this therapy. Samples from the EURTAC trial were prospectively screened for *EGFR* mutations using a combination of laboratory-developed tests (LDTs), and tested retrospectively with the cobas *EGFR* mutation test (*EGFR* PCR test). The *EGFR* PCR test results were compared to the original LDT results and to Sanger sequencing, using a subset of specimens from patients screened for the trial. Residual tissue was available from 487 (47%) of the 1044 patients screened for the trial. The *EGFR* PCR test showed high concordance with LDT results with a 96.3% overall agreement. The clinical outcome of patients who were *EGFR*-mutation detected by the *EGFR* PCR test was very similar to the entire EURTAC cohort. The concordance between the *EGFR* PCR test and Sanger sequencing was 90.6%. In 78.9% of the discordant samples, the *EGFR* PCR test result was confirmed by a sensitive deep sequencing assay. This retrospective study demonstrates the clinical utility of the *EGFR* PCR test in the accurate selection of patients for anti-*EGFR* TKI therapy. The *EGFR* PCR test demonstrated improved performance relative to Sanger sequencing.

## Introduction

The efficacy of many novel targeted cancer therapies can be predicted by the detection of specific biomarkers in the tumor. The FDA has indicated that if the identification of a specific biomarker is required for the safe and efficacious administration of a drug, a well-validated FDA approved companion diagnostic assay is required for that drug. The optimal approval path for a new targeted therapy and its companion diagnostic is a parallel clinical development process that involves clinical trials for the investigational agent where the investigational diagnostic test is used to either select patients for the trials or to predict response to treatment, and ends ideally with simultaneous health authority approval of the drug and the companion diagnostic. Successful examples of this include the co-development (and co-approval) of the BRAF inhibitor vemurafenib and its companion diagnostic BRAF V600E mutation assay for BRAF-mutant metastatic melanoma[1], and the ALK inhibitor crizotinib and its companion diagnostic ALK fusion gene test in advanced ALK-fusion positive non-small cell lung cancer (NSCLC) patients.[2], [3], [4]

However, in some cases, predictive biomarkers for a targeted therapy are not recognized until after the drug is first approved. As an example, the anti-*EGFR* antibody cetuximab was first approved in the US for the treatment of metastatic colorectal cancer in 2004. Numerous retrospective and prospective trials subsequently revealed that tumors harboring *KRAS* mutations were very unlikely to respond to cetuximab. In July 2009, FDA required labeling changes for cetuximab and another anti-*EGFR* antibody panitumumab requiring that the indications and usage state there was no treatment benefit with the drugs for patients whose tumors had *KRAS* mutations in codon 12 or 13, at a time when there were no FDA-approved diagnostic assays for *KRAS* mutations.[5] Only later, in July 2012, did a *KRAS* mutation assay receive FDA approval, based on the results of a prospective randomized trial, highlighting the challenges of retrospectively validating a companion diagnostic assay after the pivotal drug trials have been completed.[6]

The anti-*EGFR* TKI erlotinib was initially approved for all patients with advanced NSCLC who had progressed on first-line chemotherapy. A number of subsequent studies determined that patients with *EGFR*-mutant NSCLC had a high likelihood of responding to these TKI, leading to trials in the first-line setting for *EGFR*-mutant cancer.[7], [8], [9], [10], [11], [12], [13] Four prospective randomized clinical trials studied in Asian populations demonstrated that erlotinib and gefitinib resulted in improved progression-free survival compared to chemotherapy for first line therapy in NSCLC patients with *EGFR* mutations.[7], [8], [9], [13] Other clinical studies in mixed ethnicity cohorts have concluded with similar results.[10],[12]

The EURTAC trial was a randomized phase 3 trial to assess the safety and efficacy of erlotinib compared with standard platinum-based chemotherapy for first-line treatment of a patient population with advanced *EGFR*-mutation detected NSCLC in a largely Caucasian population of European patients. Erlotinib-treated patients experienced significant improvements in median PFS (9.7 months vs. 5.2 months) compared to chemotherapy. Patients on the erlotinib arm also had a considerably higher percentage of responses (58% vs. 15%) in the intent-to-treat population.[11] This trial has been submitted for first line indication of erlotinib in *EGFR* mutated NSCLC patients.

The majority of activating *EGFR* mutations are located in exons 19 (45%) and 21 (40–45%).[14], [15], [16], [17], [18], [19], [20] Guidelines from organizations such as ASCO, CAP/AMP, and NCCN recommend the use of anti-*EGFR* TKIs as first-line therapy in patients with *EGFR*-mutant advanced NSCLC based on the results of these pivotal clinical trials. [21], [22], [23] Recent recommendations by CAP/IASLC/AMP advise the identification of EGFR mutations present at >1% of which exon 19 deletions and an exon 21 mutation (L858R) account for greater than 90% of all mutations.[24] None of the guidelines specify the testing method to be used, however the cobas *EGFR* Mutation test is CE-IVD approved and is recently FDA approved.[25]

Here we present the retrospective analysis of a clinical validation study of the *EGFR* PCR test on a subset of lung cancer specimens from patients screened for the EURTAC trial. The *EGFR* PCR test demonstrated improved sample workflow relative to the LDTs used in the EURTAC trial, enabling *EGFR* mutation screening in a single assay with a one-day turn-around time. The *EGFR* PCR test showed superior sensitivity and specificity compared with conventional Sanger sequencing.

## Methods

The major study objectives were 1) to correlate the clinical outcomes (PFS, BORR) from the subgroup of available samples tested by the *EGFR* PCR test to the results from the entire EURTAC population, and 2) to compare the analytic performance of the *EGFR* PCR test to that of the original LDT and Sanger sequencing, using massively parallel pyrosequencing (MPP) to resolve discrepancies observed between the other 3 testing methods.

**Figure 1:**
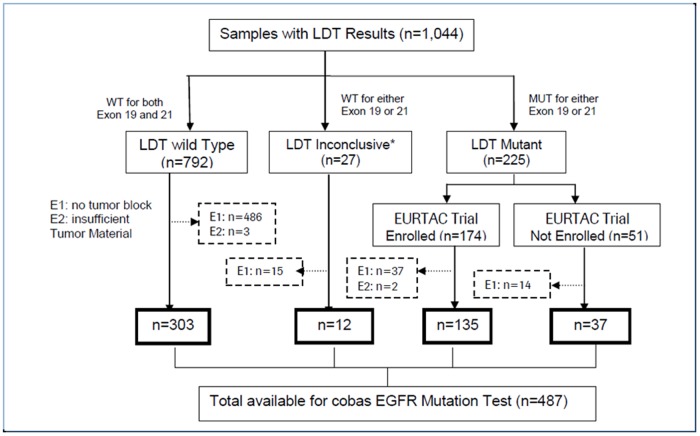
Flow of samples through the study. E1 samples: tumor block not available for analysis. E2 samples: tumor material insufficient for analysis. LDT  =  laboratory-developed test.

In the EURTAC trial, 1,044 patients from hospitals in France, Italy, and Spain were screened using the LDT. For this study, all samples were retrospectively analyzed under IRB approval from Copernicus IRB (00001313). Site specific IRB approval from each clinical site and written consent from all patients was obtained prior to the study conduct phase of NCT00446225.[11], [26] In 487 cases, residual specimens were available for retesting with the *EGFR* PCR test (Figure 1). A single 5 µm section with at least 10% tumor content from each of the 487 specimens was used for the *EGFR* PCR test. Genomic DNA from existing eluate or extracted from additional sections was tested on Sanger sequencing and MPP. Table 1 lists the demographics of the patients screened for the EURTAC trial by the LDT, sub-categorized by patients tested or not tested by the *EGFR* PCR test. Patients enrolled in the EURTAC trial were selected using a laboratory-developed test, validated by the Laboratory of Oncology (ICO-Hospital Germans Trias i Pujol, Badalona, Spain) consisting of three methodologies.[26] In this study, a single PCR-based assay for detecting *EGFR* mutations was used. Details of the analytical performance of this assay have been described previously.[27]

**Table 1:** Demographics of the patient cohort screened for EURTAC trial.

	SLCG LDT MD		SLCG LDT MND	
	*EGFR* PCR tested	*EGFR* PCRnot tested	*EGFR* PCR tested	*EGFR* PCR not tested
**Total**	172	53	303	489
Age (years), mean ± SD	64.1±10.4	62.9±10.4	61.7±10.6	61.7±10.6
**Sex, n (%)**				
Male	41 (23.8)	14 (26.4)	179 (59.1)	281 (57.5)
Female	131 (76.2)	39 (73.6)	124 (40.9)	208 (42.5)
**Race/ethnicity, n (%)**				
Caucasian	168 (97.7)	52 (98.1)	296 (97.7)	481 (98.4)
Other*	4 (2.3)	1 (1.9)	7 (2.3)	8 (1.6)
**Smoking status, n (%)**				
Never smoked	124 (72.1)	31 (58.5)	74 (24.4)	133 (27.2)
Past/currentsmoker	47 (27.3)	22 (41.5)	219 (72.3)	339 (69.3)
Unknown	1 (0.6)	0 (0.0)	10 (3.3)	17 (3.5)
Stage IIIB	13 (7.6)	2 (3.8)	17 (5.6)	40 (8.2)
Stage IV	157 (91.3)	50 (94.3)	277 (91.4)	432 (88.3)
Other*	2 (1.2)	1 (1.9)	9 (3.0)	17 (3.5)
**Histology, n (%)**				
Adenocarcinoma	156 (90.7)	47 (88.7)	266 (87.8)	407 (83.2)
BronchioalveolarCarcinoma	1 (0.6)	2 (3.8)	5 (1.7)	16 (3.3)
Other*	15 (8.7)	4 (7.5)	32 (10.6)	66 (13.5)

*Other includes subjects with no information available. LDT  =  laboratory-developed test; MD  =  mutation detected; MND  =  mutation not detected.

SLCG inconclusive (n = 27) data not shown.

### Statistical considerations

Mutation Detected (MD) was defined as the presence of either an exon 19 deletion or L858R mutation. Mutation Not Detected (MND) was defined as the absence of both exon 19 deletions and the L858R mutation. SAS/STAT® software was used for all data analysis.

### Clinical outcome study statistics

Kaplan-Meier survival curves were used to assess the PFS by treatment method (chemotherapy or erlotinib) among patients who were enrolled in the EURTAC trial and screened with the LDT as well as the subset of patients who were determined to be mutation-positive by the *EGFR* PCR test. Nonparametric log-rank test was performed to assess PFS between patients who were randomized to chemotherapy or erlotinib. The hazard ratio (chemotherapy vs. erlotinib) relative to PFS was also calculated. Best overall response was the best response recorded from the start of treatment until disease progression and BORR (Best overall response rate) was summarized with 95% confidence limits according to Pearson-Clopper methods based on investigators assessment for each treatment arm.

### Analytical performance statistics

For analytical performance, an agreement analysis was performed between the *EGFR* PCR test result and the LDT test. Mutation detection of exon 19 deletions and L858R mutations were analyzed in aggregate. Separately, the *EGFR* PCR test was also compared to Sanger sequencing and MPP by a CLIA-certified laboratory. For the agreement analyses, the positive percent agreement (PPA), negative percent agreement (NPA), and overall percent agreement (OPA) with their corresponding 95% confidence intervals (CIs) were calculated. In addition, 3-way analyses using MPP as a second reference method was performed to resolve the discrepancy results.

### Mutation testing methods

#### EGFR PCR Test

The *EGFR* PCR test (cobas *EGFR* Mutation Test, Roche Molecular Systems, Inc, Branchburg, NJ, USA) is a CE-IVD marked multiplex allele-specific PCR-based assay designed to detect 41 mutations in exons 18, 19, 20, and 21 in FFPET specimens of human NSCLC.[28] DNA is isolated using the cobas DNA Sample Preparation Kit (Roche Molecular Systems, Branchburg, NJ). [29] A minimum of 150 ng of genomic DNA is required for PCR amplification, which can typically be isolated from a single 5 µm FFPET section. The *EGFR* PCR test software version used in this study was designed to detect 29 deletions in exon 19 and 2 L858R variants in exon 21. Macrodissection is only recommended if tumor content is less than 10%; laser capture microdissection is not required. The *EGFR* PCR test was performed per manufacturer's package insert and results were automatically analyzed and reported. The limit of detection has been validated to 5% mutant alleles. The workflow from DNA isolation to results reporting can be performed in one 8 hour period.[27]

#### LDT

Patients in the EURTAC study were screened using a combination of methods developed by Laboratory of Oncology, ICO-Hospital Germans Trias i Pujol, Barcelona, Spain.[11] In short, EGFR activating mutations in exons 19 and 21 were initially identified by Sanger sequencing and confirmed by fragment length analysis for exon 19 deletions (FAM-labelled primer in an ABI prism 3130 DNA analyser (Applied Biosystems, Foster City, CA, USA) and by Taqman assay for exon 21 (L858R) mutation. All tumor specimens were from the original biopsy taken prior to any treatment and before randomization. Testing was performed on ≥ 2mm^2^ of tissue obtained from one to three slides of 4-micron tissue sections which were subjected to laser capture microdissection to enrich for the presence of tumor cells. DNA was extracted using a standard laboratory protocol and tested at a single site in Spain in Laboratory of Oncology for *EGFR* activating mutations in exon 19 and 21 using a previously described method. The average turnaround time was approximately 5 days.[26]

#### Bi-directional Sanger sequencing

All samples tested by the *EGFR* PCR test were also tested by Sanger sequencing using DNA from FFPET specimens prepared by the cobas DNA Sample Preparation Kit and sequenced with 2× bidirectional Sanger sequencing by a CLIA-certified laboratory (SeqWright, Houston, TX, USA) using a validated protocol. Repeat Sanger sequencing was performed to compare the detection of *EGFR* mutations from adjacent sections of tissue to minimize any impact of tissue heterogeneity used for the *EGFR* PCR test relative to the original LDT results. Also, sequencing protocols vary by laboratory in terms of the percent tumor content/sample that requires macrodissection. DNA isolated with the cobas DNA Sample Preparation Kit and used for sequencing required ≥10% tumor content. Average turnaround time to results was 7 days. The estimated limit of detection is approximately 20% mutant alleles.[30]

#### Massively parallel pyrosequencing (MPP)

Samples with valid *EGFR* PCR test results with adequate DNA remaining from the initial extraction were tested by a MPP method (454 GS Titanium, 454 Life Sciences, Branford, CT, USA) by a CLIA-certified laboratory (SeqWright, Houston, TX, USA) using a validated protocol.[31] This method is a 5–7 day process that involves amplicon generation, pooling, ligation, emulsion PCR, amplification and massively parallel pyrosequencing with manual data analysis. The estimated limit of detection for the assay is 1.25% mutant alleles. [27] The MPP method was used to demonstrate performance of the EGFR PCR test to a more sensitive method and as an arbiter for discrepant cases observed between the LDT or the repeat Sanger sequencing. In order to preserve patient privacy associated with tested clinical samples, raw MPP sequencing results were anonymized and presented in Table S1.

## Results

### Specimen demographics

487 (47%) of 1,044 specimens screened for the EURTAC trial using LDTs were available for testing using the *EGFR* PCR test. The flow of samples through the study is shown in Figure 1. Patient demographics and baseline tumor characteristics for all patients by LDT status are shown in Table 1. There were no significant differences between subsets of patients tested and patients not tested by the *EGFR* PCR test (p>0.05) for each LDT status (mutation detected, mutation not detected) with the exception of country of the screening clinic.

### Clinical outcomes for patients based on the EGFR PCR test results

**Figure 2:**
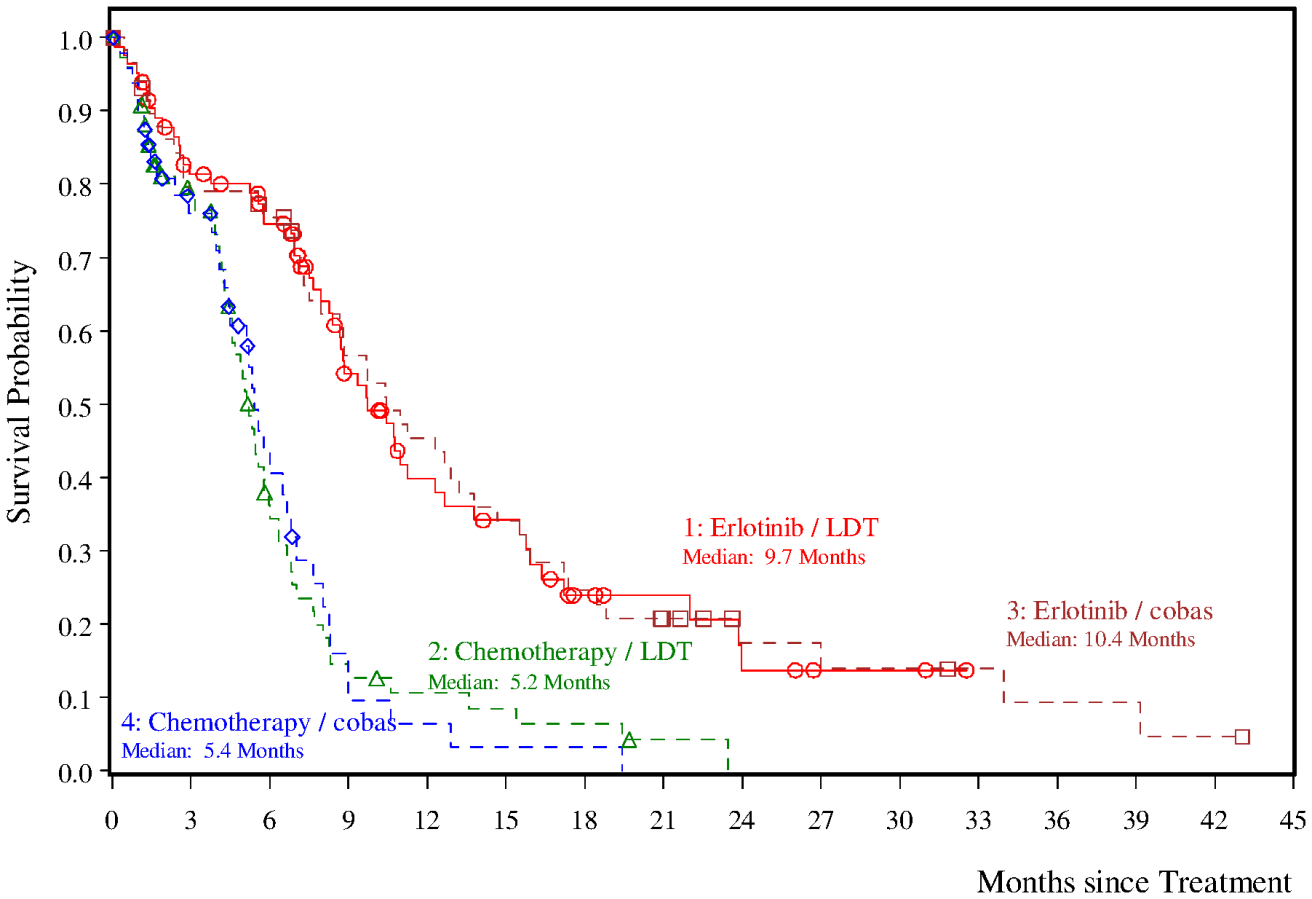
Kaplan-Meier curves of progression-free survival (PFS) for different treatments in treatment-naïve patients with non–small-cell lung cancer and *EGFR* mutation detected by the *EGFR* PCR test and LDT.

Of the 174 patients enrolled in EURTAC trial, specimens from 134 (77%) patients were available for testing using the *EGFR* PCR test. Excluding 11 patients with invalid *EGFR* PCR test results and 7 patients with a result of *EGFR* mutation not detected, a total of 116 (67%) patients were mutation detected by the *EGFR* PCR test and evaluable for clinical outcome analysis (57 patients in the chemotherapy arm and 59 in the erlotinib arm). Clinical outcomes (PFS, BORR, and OS) are presented in Table 2. Among *EGFR* PCR test positive patients, those treated with erlotinib had a significantly prolonged PFS when compared to patients treated with chemotherapy (p-value <0.0001, log-rank test); the median PFS was 10.4 months (95% CI: 8.0 to 13.8 months) and 5.4 months (95% CI: 4.4 to 6.8 months) for patients treated with erlotinib or chemotherapy, respectively (Figure 2). The HR based on the Cox proportional hazards model was reduced by 66% (HR 0.34; [95% CI: 0.21 to 0.54]) for patients in the erlotinib versus chemotherapy arm. One year after randomization, a higher percentage of patients in the erlotinib compared with the chemotherapy arm were event-free (45% [95% CI: 32% to 59% versus 6% [95% CI: 0% to 15%], respectively).

**Table 2:** Summary of Clinical Outcome Analysis among *EGFR* PCR test positive patients in the EURTAC trial.

	Chemotherapy (N = 57)	Erlotinib (N = 59)	
**PFS (Investigator)**			
Patients with event	37 (64.9%)	47 (79.7%)	
Patients without event^a^	20 (35.1%)	12 (20.3%)	
** Time to event (months)**			
Median^b^ (95%CI)	5.4 [4.4; 6.8]	10.4 [8.0; 13.8]	
p-Value (Log-Rank Test)	<0.0001		
** Hazard Ratio (**95% CI)	0.34 [0.21; 0.54]		
** 1 year estimate**			
Patients remaining at risk	2	24	
Event-free Rate^b^ (95%CI)	6% [0%; 15%]	45% [32%; 59%]	
**Best Overall Analysis**			
Response rates (95% CI)	14.0% [ 6.3%; 25.8%]	59.3%[ 45.7%; 71.9%]	
Difference in Response Rates (%)	45.29% [ 28.8%; 61.7%]		
p-Value (Chi-squared Test)	<.0001		
Odds Ratio (95% CI)	8.93 [3.59; 22.19]		
**OS**			
Patients with event	35 (61.4%)		36 (61.0%)
Patients without event^a^	22 (38.6%)		23 (39.0%)
** Time to event (months)**			
Median^b^ (95%CI)	20.8 [17.3; 29.4]		25.8 [16.1; 30.0]
p-Value (Log-Rank Test)	0.5381		
** Hazard Ratio** (95% CI)	0.86 [0.54; 1.38]		
** 2 - year estimate**			
Patients remaining at risk	16		23
Event-free Rate^b^ (95% CI)	43% [29%; 57%]		51% [38%; 64%]

Note: All eligible patients enrolled in study ML20650 were determined as *EGFR* mutation detected by the LDT. Among those, patients with *EGFR* mutation confirmed by the *EGFR* PCR test were included in this table.

Event  =  Death or progression free, whichever comes first for PFS analysis and event = death for OS analysis.

a censored.

b Kaplan-Meier estimates.

C including censored observations.

BORR were higher in patients in the erlotinib arm (59.3% [95% CI: 45.7% to 71.9%]) compared to the chemotherapy arm (14.0% [95% CI: 6.3% to 25.8%]). Patients in the erlotinib arm were much more likely to respond to therapy than patients in the chemotherapy arm (odds ratio of 8.93, [95% CI: 3.59 to 22.19]).

There was no significant difference in OS between the treatment arms (25.8 months in the erlotinib arm (95% CI: 16.1 to 30.0) and 20.8 months in the chemotherapy arm (95% CI: 17.3 to 29.4) (log-rank test p-value  = 0.5381)).

PFS, BORR and OS results for *EGFR* PCR test positive patients did not differ significantly from those obtained in all patients enrolled in the EURTAC trial which suggests that the *EGFR* PCR test positive patients are representative of all EURTAC enrolled patients.

For the 7 cases where the *EGFR* PCR test result was mutation not detected and discrepant with the LDT, two cases resolved in favor of the LDT by MPP, three cases resolved in favor of the *EGFR* PCR test and one sample was invalid for both Sanger and MPP and the other was in agreement between the *EGFR* PCR test and Sanger but not MPP (Table S2). Anecdotally, 6 of the 7 patients were treated with erlotinib and only one patient achieved greater than or equal to median PFS based on the LDT or the *EGFR* PCR test.

### Comparison of EGFR PCR test and LDT results

Among 432 specimens with valid results from both the *EGFR* PCR test and LDT, the PPA, NPA and OPA were 94.2% (146/155, CI: 89.3%, 96.9%), 97.5% (270/277, CI: 94.9%, 98.8%), and 96.3% (416/432, CI: 94.1%, 97.7%), respectively (Table 3). Thus there was a high concordance between the original LDT and *EGFR* PCR test results. Among sixteen specimens with discordant results, the *EGFR* PCR test result was confirmed by MPP in 68.8% (11/16) cases (Table S3).

**Table 3:** Agreement analysis between *EGFR* PCR test and LDT.

		SLCG LDT		Total
N = 432		Mutation detected	Mutation not detected	
***EGFR* PCR test**	**Mutation detected**	146	7	153
	**Mutation not detected**	9	270	279
	**Total**	155	277	432*

•12 samples with inconclusive LDT results and 43 samples with invalid *EGFR* PCR test results were excluded.

Positive percent agreement  = 94.2% (95% CI [89.3–96.9%]).

Negative percent agreement  = 97.5% (95% CI [94.9–98.8%]).

Overall percent agreement  = 96.3% (95% CI [94.1–97.7%]).

### Comparison of the EGFR PCR test results with Sanger Sequencing

Of 487 specimens tested using the *EGFR* PCR test and Sanger sequencing, 406 gave valid results by both methods (38 were invalid by both methods, five were invalid by *EGFR* PCR test and 38 were invalid by Sanger sequencing). The PPA, NPA and OPA for *EGFR* PCR test compared with Sanger sequencing were 96.6% (112/116, CI: 91.7%, 98.7%), 88.3% (256/290, CI: 84.1%, 91.5%), and 90.6% (368/406, CI: 87.4%, 93.1%; Table 4), respectively. Among 38 discordant results between the *EGFR* PCR test and Sanger sequencing, MPP agreed with the *EGFR* PCR test result in 30 (78.9%) cases (Table S4). Sanger sequencing detected one L858R not detected by MPP and failed to detect 22 exon 19 deletions and 7 L858R mutations confirmed by MPP. Four MPP results were invalid, and the remaining four results agreed with Sanger. The range of percent mutant alleles of the cases missed by Sanger was 3% to 60%, with several specimens (n = 16) under the estimated limit of detection for Sanger.

**Table 4:** Agreement analysis between *EGFR* PCR test and Sanger sequencing.

		Sanger sequencing		Total
N = 406		Mutation detected	Mutation not detected	
***EGFR*** ** PCR test**	**Mutation detected**	112	34	146
	**Mutation not detected**	4	256	260
	**Total**	116	290	406

*81 samples with invalid *EGFR* PCR test or Sanger sequencing results were excluded.

Positive percent agreement  = 96.6% (95% CI [91.5–98.7%]).

Negative percent agreement  = 88.3% (95% CI [84.1–91.5%]).

Overall percent agreement  = 90.6% (95% CI [87.4–93.1%]).

## Discussion

This study supports the feasibility of performing a retrospective clinical validation of a companion diagnostic from prospective, therapeutic clinical trials. The *EGFR* PCR test results were highly concordant (>96%) with the LDT results used to select patients for the EURTAC trial. As a consequence, PFS and BORR of the subset of patients with *EGFR* mutations detected with the *EGFR* PCR test were comparable to the full cohort of patients enrolled in the EURTAC trial, thus validating the use of the *EGFR* PCR test to select patients for treatment with anti-*EGFR* TKIs such as erlotinib. Median PFS survival was 9.7 versus 10.4 months for the erlotinib group and 5.2 versus 5.4 months for the LDTs and *EGFR* PCR test, respectively. The BORR was 58% versus 59.3% months for the erlotinib group and 15% versus 14.0% for the LDTs and *EGFR* PCR test, respectively. Among the 16 discordant specimens between the *EGFR* PCR test and LDTs, a third mutation testing method agreed with the *EGFR* PCR test result in 11 cases. Of seven cases that were mutation detected by the *EGFR* PCR test and mutation not detected by the LDT, 5 were confirmed by MPP. These patients could have potentially benefited from anti-*EGFR* TKI therapy. The *EGFR* PCR test had a number of technical advantages over the LDT used in the EURTAC trial. The LDT required laser capture microdissection of multiple tissue sections and involved 3 separate assays with a median turnaround time of 4.5 days. By comparison the *EGFR* PCR test required macrodissection only if the tumor content was <10% and can be performed in one day using a single 5 µm section. Furthermore the *EGFR* PCR test is a commercially available kit-based assay that provides an automated result, rather than a manual process subject to interpretation and which can be performed by any qualified clinical laboratory.

More than 80% of the specimens tested in this study were small biopsy specimens. The overall invalid rate for Sanger sequencing was 15.6% (76/487) compared to the *EGFR* PCR assay at 9% (43/487). However, the invalid rate for the subset of specimens derived from resected specimens was 0% (0/109) likely because of sufficient tissue availability. Thus the assay is extremely robust when performed on resected tumor specimens and has an approximately 90% success rate on biopsy specimens, which are often the only tumor sample available for testing in NSCLC.

Sanger sequencing has been widely used to detect *EGFR* mutations.[30], [32] Similar to the overall invalid rates, for the 134 *EGFR* mutation detected LDT samples enrolled in the EURTAC trial, Sanger sequencing had a higher invalid rate (15.7%) compared to 8.2% for the *EGFR* PCR test. There were also 30 mutation not detected results for Sanger sequencing (22.4%) and 7 mutation not detected results for the *EGFR* PCR test (5.2%). With 21 invalid results and 30 mutation not detected results, Sanger sequencing would have misclassified 38% of patients enrolled in the EURTAC trial. Similar invalid rates have been reported in three other studies, suggesting that this methodology has limitations when applied to DNA from FFPET samples.[33], [34], [35] In addition, Sanger sequencing has shown poor sensitivity in samples containing less than 20–25% mutant alleles.[35], [36], [37] When we compared the agreement between valid results for the *EGFR* PCR test with Sanger sequencing (n = 406), there were 38 discordant cases of which 30 were confirmed by MPP. Twenty-nine of the 30 cases resulted in mutation detected status by the *EGFR* PCR test and would make these patients eligible for anti-*EGFR* therapy. Poor sensitivity of Sanger sequencing thus explains the relatively low NPA compared to *EGFR* PCR test observed in this study.

Given the criticality of *EGFR* mutation testing in selecting specific therapies for life-threatening cancers such as advanced NSCLC, robust and accurate assays with rapid turnaround time are preferred. Recent quality assurance studies to ascertain the mutation status of a standard panel of tumors have shown that different clinical laboratories do not correctly identify the mutation status of 100% of the panel members, even when they are using the same or similar testing methodologies.[38], [39] For assays that involve mutation analysis of tumor samples, important factors contributing to the assay performance include analytic standardization, validation of reagents and methodology, laboratory experience, and the appropriate involvement of the pathologist.

In conclusion, results of the present study indicate that the cobas *EGFR* mutation test is a highly robust and highly accurate companion diagnostic assay to select patients for treatment with anti-*EGFR* therapies such as erlotinib.

## Supporting Information

Table S1 **Listing of MPP Result.**
(PDF)

Table S2 **Outcome from samples discrepant between the cobas EGFR PCR test and LDT that were enrolled in the clinical trial (cobas MND/LDT MD).**
(PDF)

Table S3 **Agreement results between discordant EGFR PCR and LDT tests.**
(PDF)

Table S4 **MPP results from resolution analysis of discordant specimens between EGFR PCR test and Sanger sequencing.**
(PDF)
