# Supplementary material for: Clinical Validation of a PCR Assay for the Detection of EGFR Mutations in Non–Small-Cell Lung Cancer: Retrospective Testing of Specimens from the EURTAC Trial
Source: PLoS One. 2014 Feb 25;9(2):e89518. doi: 10.1371/journal.pone.0089518 (PMC3934888; doi:10.1371/journal.pone.0089518)
Supplement: Table S1 — Listing of MPP Result. (PDF) [file pone.0089518.s001.pdf]

Listing of MPP Result

* Only Cosmic ID 6224 was defined as exon21 (L858R) positive for 454
Mutations by MPP or Sanger are reported as (% mutation; Cosmic ID) based on trial design

fake_id	454: Exon 19: Comments	454: Exon 21: Comments	454: Exon 19 MD/MND Flag	454: Exon 21 MD/MND Flag	454: Exon 19: Percent Mutant Read	454: Exon 21: Percent Mutant Read	454: Exon 19: Other, Specify	454: Exon 21: Other, Specify
1			MD	MND	(36.51; 12382)		(2239_2248>C~v2; 12382)	
2			MND	MD		(11.99; 6224)		(L858R:2573T>G; 6224)
3			MND	MD		(12.82; 6224)		(L858R:2573T>G; 6224)
4			MND	MND				
5			MND	MND				
6			MND	MD	(2.05; 6223)	(58.04; 6224)	(2235_2249del15; 6223)	(L858R:2573T>G; 6224)
7			MD	MND	(10.94; 6223), (2.56)		(2235_2249del15; 6223), (L747S:a.2240T>C)	
8			MND	MND				
9			MND	MND		(33.75; 6213)		(L861Q:2582T>A; 6213)
10			MND	MND				
11			MND	MND		-8.89		(2602del1)
12			MND	MD		(13.55; 6224)		(L858R:2573T>G; 6224)
13			MND	MND				
14			MND	MND				
15			MD	MND	-14.66		(2250_2276>AAA)	
16			MND	MD		(16.79; 6224)		(L858R:2573T>G; 6224)
17	Failed PCR	Failed PCR	Invalid	Invalid				
18			MND	MND				
19			MND	MND				
20			MND	MD		(21.22; 6224)		(L858R:2573T>G; 6224)
21			MND	MND				
22			MND	MND				
23			MND	MND		(3.62), (2.12)		(2602del1), (2509_2585del77)
24			MND	MND				
25			MND	MND				
26			MND	MND				
27			MND	MND				
28			MND	MND				
29			MND	MND				
30			MND	MND				
31			MND	MND				
32		Failed PCR	Invalid	Invalid				

33			MD	MND	(28.1; 6223), (1.37; 12382)		(2235_2249del15; 6223), (2239_2248>C~v2; 12382)	
34			MND	MND				
35			MND	MND				
36			MND	MND				
37			MND	MD		(17.87; 6224)		(L858R:2573T>G; 6224)
38			MND	MD		(66.87; 6224)		(L858R:2573T>G; 6224)
39			MD	MND	(17.41; 12384)		(2237_2255>T; 12384)	
40			MND	MND				
41			MD	MND	(64.39; 6223)		(2235_2249del15; 6223)	
42			MND	MND				
43			MD	MND	(15.26; 6225)		(2236_2250del15; 6225)	
44			MND	MND				
45			MND	MND				
46			MND	MND				
47			MND	MND				
48			MND	MD		(20.34; 6224)		(L858R:2573T>G; 6224)
49			MD	MND	(31.95; 6223)		(2235_2249del15; 6223)	
50		Failed PCR	Invalid	Invalid	(16.56; 6225)		(2236_2250del15; 6225)	
51			MND	MND				
52			MD	MND	-7.42		(2234_2251>AAT)	
53			MD	MND	(33.84; 6223)		(2235_2249del15; 6223)	
54			MND	MND				
55		Failed PCR	Invalid	Invalid				
56			MND	MND				
57			MD	MND	(29.98; 12382)		(2239_2248>C~v1; 12382)	
58			MND	MD		(37; 6224)		(L858R:2573T>G; 6224)
59			MND	MND				
60			MND	MND		(16.44; 6213)		(L861Q:2582T>A; 6213)
61			MND	MND				
62			MND	MND				
63			MND	MND				
64			MND	MND				
65			MND	MND				
66			MND	MND				
67			MND	MND				
68		Failed PCR	Invalid	Invalid				
69			MND	MND				
70			MND	MND				
71			MND	MND				
72			MD	MND	(1.87; 12382)		(2239_2248>C~v2; 12382)	

73			MND	MND				
74			MND	MND				
75			MD	MND	(11.44; 12384)		(2237_2255>T; 12384)	
76			MD	MND	(23.48; 6223)		(2235_2249del15; 6223)	
77			MND	MD	(2.65; 6223)	(16.49; 6224)	(2235_2249del15; 6223)	(L858R:2573T>G; 6224)
78			MND	MND				
79			MND	MND				
80			MND	MND				
81			MND	MD		(86.91; 6224)		(L858R:2573T>G; 6224)
82			MND	MND				
83			MND	MND				
84		Failed PCR	Invalid	Invalid				
85			MND	MND				
86			MND	MND		-1.63		(2602del1)
87			MD	MND	(37.96; 6223)		(2235_2249del15; 6223)	
88	Failed PCR		Invalid	Invalid				
89			MD	MND	(46.2; 12370)		(2240_2257del18; 12370)	
90			MND	MND				
91			MND	MND				
92			MND	MND				
93			MD	MND	(77.61; 12382)		(2239_2248>C~v2; 12382)	
94			MD	MND	(12.42; 6225)		(2236_2250del15; 6225)	
95			MND	MND				
96			MND	MND				
97			MD	MND	(6.4; 6223)		(2235_2249del15; 6223)	
98			MND	MND				
99			MND	MND				
100			MND	MD		(32.15; 6224)		(L858R:2573T>G; 6224)
101			MND	MND				
102		Failed PCR	Invalid	Invalid	(42.98; 23571), (28.56)		(2238_2252del15~v1; 23571), (2237_2253>GAA)	
103			MND	MND				
104			MD	MND	(82.47; 6223)		(2235_2249del15; 6223)	
105			MD	MND	(48.13; 6223)		(2235_2249del15; 6223)	
106			MND	MD		(5.44; 6224)		(L858R:2573T>G; 6224)
107			MND	MND				
108			MND	MND				
109			MD	MND	(21.47; 6223)		(2235_2249del15; 6223)	
110			MND	MND				
111			MND	MND				
112			MND	MND				

113			MD	MND	(69.74; 12370)		(2240_2257del18; 12370)	
114			MND	MND				
115			MND	MND				
116			MD	MND	(49.11; 6225)		(2236_2250del15; 6225)	
117		Failed PCR	Invalid	Invalid				
118			MND	MND				
119			MND	MND				
120			MND	MND				
121			MND	MND				
122			MD	MND	(22.76; 6225)		(2236_2250del15; 6225)	
123			MND	MND				
124			MND	MND				
125		Failed PCR	Invalid	Invalid	(26.7), (6.46; 23571)		(2237_2253>GAA), (2238_2252del15~v1; 23571)	
126			MD	MND	(13.37; 12370)		(2240_2257del18; 12370)	
127			MND	MND				
128			MND	MND				
129			MND	MD		(18.69; 6224)		(L858R:2573T>G; 6224)
130			MND	MND				
131			MND	MD		(21.1; 6224)		(L858R:2573T>G; 6224)
132			MND	MND				
133			MND	MD		(45.88; 6224)		(L858R:2573T>G; 6224)
134			MND	MND				
135			MD	MND	(50.97; 12383)		(2239_2251>C; 12383)	
136			MND	MD		(38.03; 6224)		(L858R:2573T>G; 6224)
137			MD	MND	(62.39; 6225)		(2236_2250del15; 6225)	
138			MND	MND				
139			MND	MND				
140			MND	MND				
141			MND	MND				
142			MND	MD		(5.98; 6224)		(L858R:2573T>G; 6224)
143			MND	MND				
144			MD	MND	(23.74; 6223)		(2235_2249del15; 6223)	
145			MND	MND				
146			MND	MND				
147			MD	MND	(33.61; 6223)		(2235_2249del15; 6223)	
148			MND	MND				
149			MND	MD		(91.16; 6224)		(L858R:2573T>G; 6224)
150			MND	MND				
151			MND	MD		(24.09; 6224)		(L858R:2573T>G; 6224)
152			MND	MND				

153			MND	MD		(56.18; 6224)		(L858R:2573T>G; 6224)
154		Failed PCR	Invalid	Invalid	(37.05; 6225)		(2236_2250del15; 6225)	
155			MND	MND				
156			MND	MND				
157			MND	MD		(70.39; 6224)		(L858R:2573T>G; 6224)
158			MD	MND	(41.21; 6223)		(2235_2249del15; 6223)	
159			MND	MND				
160			MND	MD		(36.53; 6224)		(L858R:2573T>G; 6224)
161			MND	MND				
162			MND	MND				
163			MND	MD		(3.7; 6224)		(L858R:2573T>G; 6224)
164			MND	MD		(73.29; 6224)		(L858R:2573T>G; 6224)
165			MND	MND				
166		Failed PCR	Invalid	Invalid				
167			MD	MND	(9; 6223)		(2235_2249del15; 6223)	
168			MD	MND	(30.66), (6.78; 6223)		(2236_2252>AT), (2235_2249del15; 6223)	
169			MND	MND				
170			MND	MND				
171			MND	MND				
172			MND	MND				
173			MND	MND				
174			MD	MND	(37.45; 12383), (15.92), (1.81)	(6.04; 6224)	(2239_2251>C; 12383), (2239_2251>C~v2), (2237_2251>AAC)	(L858R:2573T>G; 6224)
175	Failed PCR	Failed PCR	Invalid	Invalid				
176			MND	MD		(31.96; 6224)		(L858R:2573T>G; 6224)
177			MD	MND	(11.76; 6223), (4.25; 23571)		(2235_2249del15; 6223), (2238_2252del15~v1; 23571)	
178		Failed PCR	Invalid	Invalid				
179			MND	MND				
180			MND	MND				
181			MND	MD		(27; 6224), (12.62)		(L858R:2573T>G; 6224), (2602del1)
182			MND	MND				
183			MD	MND	(26.51; 6225)		(2236_2250del15; 6225)	
184			MND	MND		-7.49		(2547_2581del35)

185	Failed PCR	Failed PCR	Invalid	Invalid				
186			MND	MND				
187			MND	MND				
188			MND	MND				
189			MND	MND				
190			MND	MND				
191			MND	MD		(30.31; 6224), (3.17)		(L858R:2573T>G; 6224), (2602del1)
192			MND	MND				
193			MND	MND				
194			MND	MND				
195			MND	MND				
196			MND	MND				
197			MND	MND				
198			MND	MND				
199			MND	MND				
200			MD	MND	(31.09; 6255)		(2239_2256del18~v2; 6255)	
201			MD	MND	(30.81; 6223)		(2235_2249del15; 6223)	
202			MND	MND				
203		Failed PCR	Invalid	Invalid				
204			MND	MND				
205			MND	MND				
206			MND	MND				
207			MD	MND	(77.13; 6223)		(2235_2249del15; 6223)	
208			MND	MD		(17.35; 6224)		(L858R:2573T>G; 6224)
209			MND	MND				
210			MND	MND				
211			MND	MND				
212			MND	MND				
213			MND	MND				
214			MND	MND		-2.39		(2602del1)
215			MND	MD		(53.77; 6224)		(L858R:2573T>G; 6224)
216			MND	MD		(31.84; 6224)		(L858R:2573T>G; 6224)
217			MND	MND				
218			MND	MND				
219			MND	MND				
220			MND	MND				
221		Failed PCR	Invalid	Invalid				
222			MND	MND				
223			MND	MND				
224			MND	MND				
225			MD	MND	(37.45; 6223)		(2235_2249del15; 6223)	
226			MD	MND	(2.25; 6225)		(2236_2250del15; 6225)	
227			MND	MND				
228			MND	MND				
229			MND	MND				
230			MND	MND				

231			MND	MND				
232			MND	MND				
233			MND	MND				
234			MND	MND				
235			MND	MD		(54.02; 6224)		(L858R:2573T>G; 6224)
236			MND	MND				
237			MND	MD		(12.89; 6224)		(L858R:2573T>G; 6224)
238			MD	MND	(45.15; 6223)		(2235_2249del15; 6223)	
239		Failed PCR	Invalid	Invalid				
240			MND	MD		(56.49; 6224)		(L858R:2573T>G; 6224)
241			MND	MD		(41.27; 6224)		(L858R:2573T>G; 6224)
242			MD	MND	(14.02; 18427)		(2237_2257>TCT; 18427)	
243			MND	MND				
244			MND	MD		(33.38; 6224)		(L858R:2573T>G; 6224)
245			MND	MND				
246			MD	MND	(37.36; 6223)		(2235_2249del15; 6223)	
247			MND	MND				
248	Failed PCR	Failed PCR	Invalid	Invalid				
249			MND	MND				
250			MND	MND				
251			MD	MND	(14.16; 12382), (11.39; 12382)		(2239_2248>C~v2; 12382), (2239_2248>C~v1; 12382)	
252			MD	MND	(37.5; 6225)		(2236_2250del15; 6225)	
253			MND	MND				
254			MD	MND	(9.62; 12370)		(2240_2257del18; 12370)	
255			MD	MND	(3.29; 12384)		(2237_2255>T; 12384)	
256	Failed PCR	Failed PCR	Invalid	Invalid				
257			MND	MND				
258			MD	MND	(48.38; 6223)		(2235_2249del15; 6223)	
259			MND	MD		(6.4; 6224)		(L858R:2573T>G; 6224)
260			MND	MND				
261			MND	MND				
262			MND	MND				
263		Failed PCR	Invalid	Invalid				
264			MND	MND				
265			MND	MND				
266			MD	MND	(18.03; 6223)		(2235_2249del15; 6223)	
267			MND	MND				
268			MND	MND				

269		Failed PCR	Invalid	Invalid	(41.42; 6225)		(2236_2250del15; 6225)	
270			MD	MND	(24.14), (16.04), (7.77)		(2236_2244del9), (2236_2263>GAAGCAT~v1), (2236_2263>GAAGCAT~v2)	
271			MND	MND				
272			MND	MND				
273			MD	MND	(43.82; 12382)		(2239_2248>C~v2; 12382)	
274			MND	MND				
275			MND	MND				
276			MD	MND	(6.21; 12416), (3.72; 12416)		(2237_2253>TTGCT~v2; 12416), (2237_2253>TTGCT~v3; 12416)	
277			MND	MND				
278			MND	MND				
279			MND	MND				
280			MND	MND				
281			MD	MND	(32.23; 6223)		(2235_2249del15; 6223)	
282			MND	MND				
283			MND	MND				
284			MD	MND	(6.96; 12383), (3.85)	-1.46	(2239_2251>C; 12383), (2239_2251>C~v2)	(2602del1)
285			MND	MND				
286			MD	MND	(34.67; 6223)		(2235_2249del15; 6223)	
287			MND	MD	(17.07; 6223)	(99.37; 6224)	(2235_2249del15; 6223)	(L858R:2573T>G; 6224)
288			MND	MND				
289			MND	MND				
290		Failed PCR	Invalid	Invalid	(30.25; 6223)		(2235_2249del15; 6223)	
291			MND	MND				
292			MND	MND				
293			MD	MND	(5.82; 6223), (4.1; 12370)		(2235_2249del15; 6223), (2240_2257del18; 12370)	
294			MND	MND				
295			MND	MND				
296		Failed PCR	Invalid	Invalid				
297			MD	MND	(78.14; 12382)		(2239_2248>C~v2; 12382)	
298			MD	MND	(7.33; 12370)		(2240_2257del18; 12370)	
299			MND	MND				
300			MND	MD		(71.61; 6224)		(L858R:2573T>G; 6224)
301			MND	MND				
302			MND	MND				

303			MD	MND	(13.38; 6225)		(2236_2250del15; 6225)	
304			MD	MND	(16.17; 23571)		(2238_2252del15~v1; 23571)	
305			MND	MND				
306			MND	MND				
307			MD	MND	(7.42; 6225)		(2236_2250del15; 6225)	
308			MND	MND				
309			MND	MND				
310			MND	MND				
311			MD	MND	(52.94; 12370)		(2240_2257del18; 12370)	
312			MND	MND				
313			MD	MND	(5.97; 6225)		(2236_2250del15; 6225)	
314			MND	MND				
315			MND	MND				
316			MND	MND				
317		Failed PCR	Invalid	Invalid	(76.82; 13551)		(2235_2252>AAT; 13551)	
318			MND	MND				
319			MND	MND				
320			MD	MND	(43.77; 6225)		(2236_2250del15; 6225)	
321			MD	MND	(6.76; 12370)		(2240_2257del18; 12370)	
322			MND	MND				
323			MD	MND	-30.61		(2236_2244del9)	
324			MND	MND				
325			MND	MND				
326			MND	MND				
327			MD	MND	(9.49; 12370)		(2240_2257del18; 12370)	
328			MND	MND				
329			MND	MND				
330			MND	MND				
331			MND	MND				
332			MND	MD		(67.06; 6224)		(L858R:2573T>G; 6224)
333			MD	MND	(70.45; 12382)		(2239_2248>C~v2; 12382)	
334			MD	MND	(28.79; 6223)		(2235_2249del15; 6223)	
335			MND	MD		(66.2; 6224)		(L858R:2573T>G; 6224)
336			MND	MND				
337			MND	MND				
338			MND	MND				
339			MND	MND				
340			MND	MND				
341	Failed PCR	Failed PCR	Invalid	Invalid				
342			MD	MND	(32.43; 12384)		(2237_2255>T; 12384)	
343			MD	MND	(10.25; 6225)		(2236_2250del15; 6225)	
344			MND	MND				

345		Failed PCR	Invalid	Invalid				
346			MND	MND				
347			MND	MND				
348			MND	MND				
349			MND	MND				
350			MND	MND				
351			MND	MND				
352			MND	MD		(2.17; 6224)		(L858R:2573T>G; 6224)
353			MND	MND				
354			MND	MD		(84.49; 6224)		(L858R:2573T>G; 6224)
355			MND	MD		(4.6; 6224)		(L858R:2573T>G; 6224)
356			MD	MND	(3.61; 6223)		(2235_2249del15; 6223)	
357			MD	MND	(55.81; 6223)	(9.34; 6224)	(2235_2249del15; 6223)	(L858R:2573T>G; 6224)
358			MND	MND				
359			MND	MD		(33.63; 6224)		(L858R:2573T>G; 6224)
360			MD	MND	(36.06; 6223)		(2235_2249del15; 6223)	
361			MND	MND				
362			MND	MND				
363			MND	MND				
364			MD	MND	(44.41; 6223)		(2235_2249del15; 6223)	
365			MND	MND				
366			MND	MD		(68.42; 6224)		(L858R:2573T>G; 6224)
367			MND	MND				
368			MD	MND	(25.15; 23571)		(2238_2252del15~v1; 23571)	
369			MND	MND				
370			MD	MND	(38.34; 6255)		(2239_2256del18~v2; 6255)	
371			MD	MND	(43.65; 6223)		(2235_2249del15; 6223)	
372			MD	MND	(5.43; 6223)		(2235_2249del15; 6223)	
373			MND	MND				
374			MND	MND				
375			MND	MND				
376			MND	MD		(19.11; 6224)		(L858R:2573T>G; 6224)
377			MND	MND				
378			MND	MND				
379			MD	MND	(41.57; 6223)		(2235_2249del15; 6223)	
380			MND	MND				
381			MD	MND	(15.47; 12384)		(2237_2255>T; 12384)	
382			MD	MND	(41.73; 12383), (15.81)		(2239_2251>C; 12383), (2237_2251>AAC)	

383			MD	MND	-53.46		(2239_2253>CAA)	
384		Failed PCR	Invalid	Invalid				
385			MND	MND				
386			MD	MND	(52.64; 6225)		(2236_2250del15; 6225)	
387			MND	MND				
388			MND	MND				
389			MD	MND	(17.86; 12383), (5.8), (4.66)		(2239_2251>C; 12383), (2239_2251>C~v2), (2237_2251>AAC)	
390			MND	MD		(12.73; 6224)		(L858R:2573T>G; 6224)
391			MND	MND				
392			MND	MND				
393			MD	MND	(14.74; 12384)		(2237_2255>T; 12384)	
394		Failed PCR	Invalid	Invalid	(8.09; 23571)		(2238_2252del15~v1; 23571)	
395			MND	MND				
396			MND	MND				
397			MD	MND	(38.27), (15.01)		(2237_2253>AACCT), (2239_2253>CCT)	
398			MND	MND				
399			MND	MD		(24.04; 6224)		(L858R:2573T>G; 6224)
400		Failed PCR	Invalid	Invalid				
401			MND	MND				
402			MND	MND				
403			MND	MND				
404			MND	MND				
405			MND	MND				
406			MND	MND				
407			MND	MND				
408			MD	MND	(21.08; 6223)		(2235_2249del15; 6223)	
409			MND	MND				
410			MD	MND	(76.12; 23571)		(2238_2252del15~v1; 23571)	
411		Failed PCR	Invalid	Invalid				
412			MD	MND	(26.55; 23571)		(2238_2252del15~v1; 23571)	
413			MD	MND	(35.3; 6225)		(2236_2250del15; 6225)	
414			MND	MND				
415			MD	MND	(43.98; 6223)		(2235_2249del15; 6223)	
416			MND	MND				
417			MND	MND				
418			MND	MND				
419			MND	MND				

420			MND	MND				
421			MD	MND	(2.43; 6223)		(2235_2249del15; 6223)	
422			MND	MND				
423			MD	MND	-49.27		(2239_2257>GT)	
424	Failed PCR	Failed PCR	Invalid	Invalid				
425			MND	MND				
426			MND	MND				
427			MD	MND	(14.95; 6225)		(2236_2250del15; 6225)	
428			MND	MND				
429			MND	MD		(10.32; 6224)		(L858R:2573T>G; 6224)
430			MND	MND				
431		Failed PCR	Invalid	Invalid				
432			MND	MND				
433		Failed PCR	Invalid	Invalid				
434	Failed PCR	Failed PCR	Invalid	Invalid				
435			MND	MND		(3.1; 6213)		(L861Q:2582T>A; 6213)
436			MND	MND				
437			MND	MND				
438		Failed PCR	Invalid	Invalid	(18.31; 12382)		(2239_2248>C~v2; 12382)	
439			MND	MND				
440			MND	MD	(6.78; 6255)	(49.32; 6224)	(2239_2256del18~v2; 6255)	(L858R:2573T>G; 6224)
441			MND	MD		(62.2; 6224)		(L858R:2573T>G; 6224)
442			MD	MND	(9.16; 12384), (2.71; 6223)		(2237_2255>T; 12384), (2235_2249del15; 6223)	
443			MD	MND	(24.58; 6223)		(2235_2249del15; 6223)	
444			MND	MND				
