# Supplementary material for: Clinical Validation of a PCR Assay for the Detection of EGFR Mutations in Non–Small-Cell Lung Cancer: Retrospective Testing of Specimens from the EURTAC Trial
Source: PLoS One. 2014 Feb 25;9(2):e89518. doi: 10.1371/journal.pone.0089518 (PMC3934888; doi:10.1371/journal.pone.0089518)
Supplement: Table S2 — Outcome from samples discrepant between the cobas EGFR PCR test and LDT that were enrolled in the clinical trial (cobas MND/LDT MD). (PDF) [file pone.0089518.s002.pdf]

Table S2: Outcome from samples discrepant between the cobas EGFR PCR test and LDT that were enrolled in the clinical trial (cobas MND/LDT MD)

Sample	Randomization Arm	PFS Censor Status	PFS INV (months)	OS censor status	Overall Survival (months)	Best Overall Response	cobas	LDT	Sanger	MPP
1	Chemotherapy	PD/Death	6.3	Censor	25.3	Partial Response	MND	MD	MND	MD
2	Erlotinib	PD/Death	2.6	Death	3.3	Stable Disease	MND	MD	MD	MD
3	Erlotinib	PD/Death	0.1	Death	0.1		MND	MD	MND	MND
4	Erlotinib	PD/Death	1.3	Death	21.9	Progressive Disease	MND	MD	Invalid	Invalid
5	Erlotinib	Censor	30.8	Censor	33.4	Complete Response	MND	MD	MD	MD
6	Erlotinib	PD/Death	8.7	Death	8.7	Partial Response	MND	MD	MND	MND
7	Erlotinib	PD/Death	4.2	Censor	17.1	Stable Disease	MND	MD	MND	MND
