# Supplementary material for: Clinical Validation of a PCR Assay for the Detection of EGFR Mutations in Non–Small-Cell Lung Cancer: Retrospective Testing of Specimens from the EURTAC Trial
Source: PLoS One. 2014 Feb 25;9(2):e89518. doi: 10.1371/journal.pone.0089518 (PMC3934888; doi:10.1371/journal.pone.0089518)
Supplement: Table S3 — Agreement results between discordant EGFR PCR and LDT tests. (PDF) [file pone.0089518.s003.pdf]

Table S3: Agreement results between discordant *EGFR* PCR and LDT tests

Obs	ID	cobas	LDT	MPP	Sanger	MPP_codon19	MPP_codon21	Sanger_codon19	Sanger_codon21
1	TQ0901	MD	MND	MD	Invalid	(36.51; 12382)			
2	TQ0551	MD	MND	MD	MND		(11.99; 6224)		
3	TQ1067	MD	MND	MD	MD		(12.82; 6224)		CTG/CGG;6224
4	TQ0755	MD	MND	MND	MD				CTG/CGG;6224
5	TQ0751	MD	MND	MND	Invalid				
6	TQ0833	MD	MND	MD	MD	(2.05; 6223)	(58.04; 6224)		CTG/CGG;6224
7	TQ0077	MD	MND	MD	MND	(10.94; 6223), (2.56)			
8	TQ1173	MND	MD	MND	MND				
9	TQ0911	MND	MD	MND	MND		(33.75; 6213)		
10	TQ0546	MND	MD	Invalid	Invalid				
11	TQ0343	MND	MD	MND	MND				
12	TQ0720	MND	MD	MND	MND		(8.89; 2602del1)		
13	TQ0865	MND	MD	MD	MND		(13.55; 6224)		
14	TQ0761	MND	MD	MND	MND				T/A;6213
15	TQ0286	MND	MD	MND	MND				
16	TQ0148	MND	MD	MD	MD	(14.66; 2250_2276>AAA)			
17	TQ0560	MND	MD	MD	MD		(16.79; 6224)		CTG/CGG;6224

Only Cosmic ID 6224 was defined as exon21 (L858R) positive for both Sanger and 454

Mutations by MPP or Sanger are reported as (% mutation; Cosmic ID) based on trial design
