# Supplementary material for: Clinical Validation of a PCR Assay for the Detection of EGFR Mutations in Non–Small-Cell Lung Cancer: Retrospective Testing of Specimens from the EURTAC Trial
Source: PLoS One. 2014 Feb 25;9(2):e89518. doi: 10.1371/journal.pone.0089518 (PMC3934888; doi:10.1371/journal.pone.0089518)
Supplement: Table S4 — MPP results from resolution analysis of discordant specimens between EGFR PCR test and Sanger sequencing. (PDF) [file pone.0089518.s004.pdf]

Table S4: MPP results from resolution analysis of discordant specimens between *EGFR* PCR test and Sanger sequencing

	***EGFR* PCR test**	**Sanger sequencing**	**No.**	**MPP**
1	MND	Exon 19 deletion	1	Exon 19 deletion2250_2276>AAA
2	MND	Exon 19 deletion	1	Exon 19 deletion2235_2249del15
3	MND	Exon 21L858R	1	Exon 21 L858R
4	MND	Exon 21L858R	1	MND
**Total**			**4**	

	***EGFR* PCR test**	**Sanger sequencing**	**No.**	**MPP**
1	Exon 19 deletion	MND	5	Exon 19 deletion2236_2250del15
2	Exon 19 deletion	MND	6	Exon 19 deletion2235_2249del15
3	Exon 19 deletion	MND	4	Exon 19 deletion2239_2256del18
4	Exon 19 deletion	MND	2	Exon 19 deletion2237_2255>T
5	Exon 19 deletion	MND	1	Exon 19 deletion2234_2251>AAT
6	Exon 19 deletion	MND	1	Exon 19 deletion2236_2252>AT
7	Exon 19 deletion	MND	1	Exon 19 deletion 2237_2253>TTGCT
8	Exon 19 deletion	MND	1	Exon 19 deletion2239_2251>C
9	Exon 19 deletion	MND	1	Exon 19 deletion2239_2256del18

10	Exon 21 L858R	MND	7	Exon 21 L858R
11	Exon 21 L858R	MND	1	MND
12	Exon 21 L858R	MND	4	Invalid
**Total**			**34**	

MD = mutation detected; MND = mutation not detected.
